# Elastic and Dynamic Heterogeneity in Aging Alginate Gels

**DOI:** 10.3390/polym13213618

**Published:** 2021-10-20

**Authors:** Raffaele Pastore, Ciro Siviello, Domenico Larobina

**Affiliations:** 1 Department of Chemical, Materials and Production Engineering, University of Napoli “Federico II”, Piazzale Tecchio 80, 80125 Napoli, Italy; raffaele.pastore@unina.it; 2 Institute of Polymers, Composites, and Biomaterials, National Research Council of Italy Piazzale E. Fermi 1, 80055 Portici, Italy; ciro.siviello@ipcb.cnr.it

**Keywords:** physical gels, alginate, elastic heterogeneity, dynamic heterogeneity

## Abstract

Anomalous aging in soft glassy materials has generated a great deal of interest because of some intriguing features of the underlying relaxation process, including the emergence of “ultra-long-range” dynamical correlations. An intriguing possibility is that such a huge correlation length is reflected in detectable ensemble fluctuations of the macroscopic material properties. We tackle this issue by performing replicated mechanical and dynamic light scattering (DLS) experiments on alginate gels, which recently emerged as a good model-system of anomalous aging. Here we show that some of the monitored quantities display wide variability, including large fluctuations in the stress relaxation and the occasional presence of two-step decay in the DLS decorrelation functions. By quantifying elastic fluctuation through the standard deviation of the elastic modulus and dynamic heterogeneities through the dynamic susceptibility, we find that both quantities do increase with the gel age over a comparable range. Our results suggest that large elastic fluctuations are closely related to ultra-long-range dynamical correlation, and therefore may be a general feature of anomalous aging in gels.

## 1. Introduction

Physical macromolecular gels are characterized by a network of long polymers bonded by reversible cross-links. These bonds can break and reform under the action of thermal agitation and/or stress, as in colloidal gels [1], leading to a rearrangement of the overall network on larger time- and length-scales. The ability to rearrange makes physical gels, whether colloidal or macromolecular, different from chemical gels, in which bonds are permanent, and the network structure is essentially frozen. In most situations, physical gels are prepared far from equilibrium and the rearrangement dynamics leads to irreversible evolution of the structure, which is known as “aging” [2].

During recent decades, a body of experimental observations, mainly based on DLS or similar techniques, have revealed that the aging of many physical gels, as well as of other soft amorphous solids, display distinctive relaxation dynamics, with compressed exponential decay of the auto-correlation functions and a ballistic wave-vector dependence displayed by their characteristic time [3,4,5,6,7,8,9,10,11,12,13]. These benchmarks led to the coining of the term “anomalous aging” [14,15] to classify this dynamics, highlighting its difference from sluggish, slower-than-exponential [16,17,18,19,20,21,22,23], or simple exponential relaxation (at long time) [24,25], which is paradigmatic of aging glassy materials. Understanding the microscopic origin of such anomalous aging has raised a great deal of interest during the last few years. The emerging picture suggests that anomalous aging gels are essentially athermal systems (i.e., their bonds are strong enough to be hardly broken by thermal fluctuations) [26], mainly rearranging because of intermittent releases of “internal stress”, which are spontaneously generated during the gelation process and locally encapsulated within the network [4,7,11,14,15,26,27,28]. The rearrangements generated by internal stresses propagate over very large distances, driven by the elasticity of the network and poorly disturbed by thermal fluctuations. Such a picture draws on the observation of dynamic heterogeneities (DHs) characterized by an “ultra-long” correlation length, eventually comparable with the system size. Such ultra-long-range correlations were first detected through spatially resolved scattering experiments on colloidal gels [7,29] and were recently reproduced by numerical simulations [28], emerging as another striking benchmark of anomalous aging. In this context, we recently showed that strontium alginate gel is a good model system of anomalous aging [5]. In particular, an indirect estimation of the length scale associated with DHs supported the existence of ultra-long correlations, ranging up to one hundred microns (three orders of magnitude larger than the typical pore size measured in similar systems [30]).

At the same time, a body of results on amorphous solids demonstrated that DHs are closely correlated with elastic heterogeneities (EHs) [20,31,32,33,34,35,36], with both DHs and EHs displaying a similar correlation length.

The central question here is whether these heterogeneities may give rise to macroscopic effects in the case of anomalous aging gels. As a matter of fact, in systems such as equilibrium supercooled liquids, the size of the dynamic heterogeneities is found to be relatively small, i.e., typically limited to tens of particles [37]: direct macroscopic implications are therefore not observed, as a macroscopic measurement in fact implies an average over a large number of correlated domains distributed within the sample. Conversely, in anomalous aging gels, heterogeneities are ultra-long-ranged and only a few domains may be present in a macroscopic sample [28], raising the intriguing possibility that macroscopic consequences of heterogeneities might be clearly detectable in this type of system.

If this is the case, some macroscopic observables, including mechanical properties, should display large ensemble fluctuations. In other words, replicating the same experiments on different samples should result in a large variability of the measurements. The goal for the work presented here was to investigate this possibility by directly probing mechanical fluctuations in an anomalous aging gel, and comparing them with DHs. To this aim, we performed replicated mechanical and DLS tests on strontium alginate gels on aging, quantifying elastic fluctuations through the standard deviation of the elastic modulus and measuring the dynamic susceptibility, a popular and quite direct probe of DH in soft glassy materials [6,38,39,40]. In order to directly probe the system structure, dynamic and mechanical tests were complemented by scanning electron microscopy (SEM).

We show that the macroscopic elasticity indeed displays a large variability, which increases with aging just as dynamic heterogeneities do. Overall, our results suggest that ultra-long-range heterogeneities have implications on the macroscopic properties of anomalous aging gels.

## 2. Materials and Methods

In this contribution, we extended our experimental campaign on strontium alginate gels, both replicating previous experiments and performing completely new measurements [5]. In particular, we duplicated the stress relaxation and the DLS experiments described in [5]. In addition, we performed new stress-strain measurements by replicating the experiments on twelve samples at each of the six considered waiting times. Finally, we also performed SEM measurements on super-critical carbon dioxide (sc-CO_2_) dried aerogel. Details on the investigated materials and on the aforementioned experimental methods are provided below.

### 2.1. Gel Preparation

MANUGEL GHB (FMC BioPolymers UK Ltd. Girvan, Ayrshire, KA269JN, UK) provided the alginate powder used in our work. According to the manufacturer’s specifications, the powder has an average molecular weight of 97,000 Da and a guluronic acid content of 63%. Previously conducted proton nuclear magnetic resonance (H-NMR) analysis confirmed the composition of our sample. The details of the NMR analysis can be found in [5]. Strontium chloride, disodium salt of ethylene-diamine-tetra-acetic acid (Na_2_-EDTA) and D-glucono-δ-lactone (GDL) were all purchased from Merk Life Science S.r.l. Milan ITALY and were used as received.

To prepare the strontium alginate gels used in our work, we used a two-stage procedure. [41,42] In the first stage, named “internal gelation”, the Sr^++^ was initially bound with EDTA are then released inside an aqueous solution of alginate at 2% w/w. The release took place through cleavage, with a slow hydrolyzing sugar (GDL), the stable Sr-EDTA complex. The high affinity of the released strontium ions for the alginate chains triggered the formation of Sr-Alg complexes, which led to the sol-gel transition. The gelation was allowed to continue for 24 h inside a Petri dish. In the second stage, termed “conditioning”, the pre-gel was immersed in a SrCl_2_ solution at a concentration equal to 5 mM and left to age for a known amount of time. After aging, the gel disc was cored and two smaller discs were taken for rheological and DLS tests. It is worth noting that the entire protocol allowed us to test two samples that were as similar as possible, i.e., obtained from the same reaction batch and which had undergone the same conditioning phase.

### 2.2. Dynamic Light Scattering

Multi-speckle dynamic light scattering (DLS), in the small-angle scattering configuration, was used to probe the dynamics on a microscopic scale [43,44], with the set-up adopted in [5] (see Figure S1 in the Supplementary Materials). It is useful to mention that the diameter of the laser beam was approximately ϕ≈5 mm, as obtained from the characteristics of the pigtailed fiber and the focal length of the collimation lens. Thus, the sample volume illuminated by the laser was $\left(\frac{3.14\cdot \phi^{2}}{4}\right)\delta \approx 39\;{\mathrm{mm}}^{3}$, where δ≈2 mm is the thickness of the sample. The sample disks have a diameter of about 20 mm. It is worth noting that sample disks used in the rheological tests had approximately the same shape and size.

In the multi-speckle DLS, the intensity from each pixel was self-correlated over time and averaged among all pixels belonging to the same scattering vector. Specifically, we correlated two images of the scattered light taken at times t and t+τ as:

(1) $$c_{I}\left(t,\tau ,\vec{q}\right)=\frac{{\langle I}_{p}\left(t\right)I_{p}{(t+\tau )\rangle }_{p}}{{\langle I}_{p}{\left(t\right)}_{p}I_{p}{(t+\tau )\rangle }_{p}}-1$$

where $I_{p}\left(t\right)$ is the intensity at time *t* of the *p*-th pixel belonging to the scattering vector $\vec{q}$, and ${\langle \cdot \rangle }_{p}$ denotes an average over the pixels of the same *q*-vector. In order to enhance the signal-to-noise ratio, $c_{I}$ is averaged over t, thus obtaining the usual intensity correlation function $g_{2}\left(\tau ,\vec{q}\right)-1$. For our particular setup, the modulus of the *q*-vectors varied between 0.3 and 4.4 ${\mu m}^{-1}$ (the corresponding length-scale λ≡2πq ranged between 1.4 and 21 μm).

For each scattering vector, the intensity correlation curves were interpolated with a single Equation (2a) or a double Equation (2b) compressed exponential function, depending on the case at hand

(2a) $$g_{2}\left(\tau ,q\right)-1=B\left(q\right)+{\left(\sqrt{1-B\left(q\right)}exp\left[-{\left(\frac{\tau }{\tau_{l}\left(q\right)}\right)}^{\beta_{l}\left(q\right)}\right]\right)}^{2}$$

(2b) $$g_{2}\left(\tau ,q\right)-1=B\left(q\right)+{\left(A\left(q\right)exp\left[-{\left(\frac{\tau }{\tau_{e}\left(q\right)}\right)}^{\beta_{e}\left(q\right)}\right]+\left(\sqrt{1-B\left(q\right)}-A\left(q\right)\right)exp\left[-{\left(\frac{\tau }{\tau_{l}\left(q\right)}\right)}^{\beta_{l}\left(q\right)}\right]\right)}^{2}$$

In Equation (2), B represents the baseline, $\tau_{e},\;\beta_{e}$ and $\tau_{l}$,$\;\beta_{l}$ the decay time and compressed exponent of the early- and late-decay of the field correlation function ($g_{1}$), and A represents the relative amplitude of the early-decay.

The characteristic time of a compressed exponential decay was evaluated as an average integral value, using the expression:

(3) $$\langle \tau \rangle \equiv \frac{\tau }{\beta }\Gamma \left(\frac{1}{\beta }\right)$$

where 〈τ〉 is the mean value and Γ(·) is the gamma function.

### 2.3. Rheological Tests

In stress relaxation tests a gel disk of 2 mm in thickness and 20 mm in diameter is glued on both plates of a Thermo Scientific Haake Mars III rheometer and measured in time after imposing a step strain. To prevent the dehydration of the sample, the lateral surface of the gel is placed in contact with a 5 mM SrCl_2_ conditioning solution. The sample is then left to equilibrate for 2 min before applying a 3% strain. We have already checked this range of strain to be in the linear regime, at least at the small–medium time scale, for calcium [45] and strontium [5], by performing consecutive stress relaxation in compression and torsion on the same sample. The stress is monitored for about 2 h since the application of the strain.

### 2.4. Compression Tests

In stress-strain tests a gel cylinder of about 5 mm in thickness and 20 mm in diameter was compressed in a force-controlled system with a dynamic mechanical apparatus (DMA TA instrument Q800). Generally, a ramp force of 1 N/min going from 10^−3^ N (initial preload) up to 0.1 N was applied, while the resulting displacement was recorded. To prevent dehydration, all samples were kept moist during the test. The modulus was evaluated from the initial slope of the stress vs. stretch ratio curve:

(4) E0≡dσxxd(l/l0)|l/l0=1

where $\sigma_{xx}$ is the stress along the compressional axis, whereas $l_{0}$ and l represent the height of the sample before and after compression.

### 2.5. Scanning Electron Microscopy

Samples of alginate aerogel were obtained via sc-CO_2_ extraction according to the following procedure. The alginate hydrogel was first subjected to solvent exchange with alcohol, in order to remove water from the alginate structure. The alcogel was then inserted into the extraction vessel, filled with sc-CO_2_ at 35 °C up to 200 bars, and dried for 2 h by flowing sc-CO_2_ at 1 kg/h. A final depressurization step of about 1 h was used to bring the vessel back to atmospheric pressure and recover the aerogel. To prepare for SEM analysis, the aerogel sample was fractured using liquid nitrogen and then sputter-coated with gold at 30 mA for 3 min. SEM of the sample surface was performed using a FEI Quanta 200 FEG microscope.

## 3. Results and Discussion

### 3.1. Mechanical Tests

#### 3.1.1. Stress Relaxation

**Figure 1:**
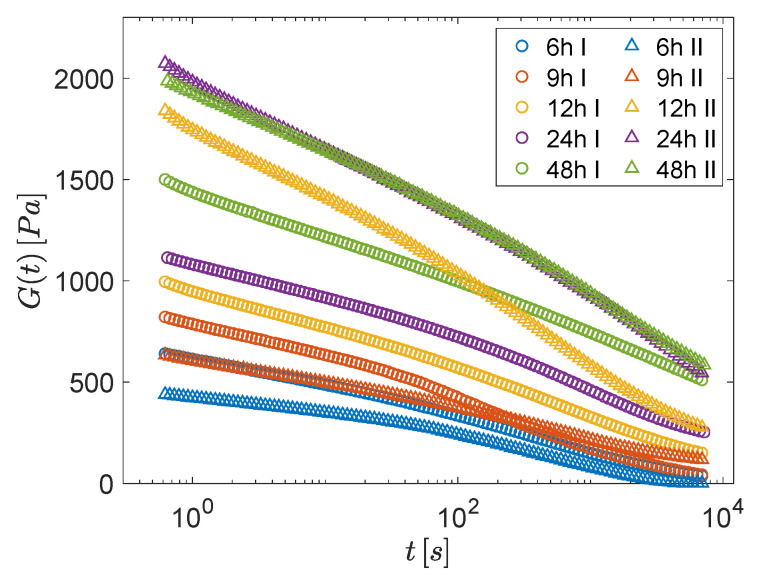
Torsional relaxation modulus vs. time for samples conditioned at different waiting times: 6 (blue), 9 (red), 12 (yellow), 24 (purple) and 48 h (green).

Figure 1 depicts duplicated stress-relaxation experiments, showing the relaxation modulus as a function of time, at five different waiting times (i.e., 6, 9, 12, 24, and 48 h).

The time-dependence of the modulus was always well described by a combination of two relaxation processes, consistently with previous results [5]: an early logarithmic decay was followed by a stretched exponential behavior over a long time [see Supplementary Materials (SM) for details on fitting and the extrapolated parameters]. The characteristic times $\tau_{o}$ and $\tau_{s}$ associated with these two relaxation processes increase with the waiting time, being compatible with different power-laws, $\tau_{o}\propto t_{w}$ and $\langle \tau_{s}\rangle \propto t_{w}^{2}$, as reported in Figure S3. Conversely, it is evident from Figure 1 that the initial values of the modulus and of the decay rates did not show a clear trend with the waiting time, as datasets corresponding to the same waiting time displayed macroscopic fluctuations and, overall, appeared quite scattered. Such macroscopic variations in the mechanical response may be interpreted as a first sign of the presence of large EHs.

#### 3.1.2. Stress-Strain Tests

**Figure 2:**
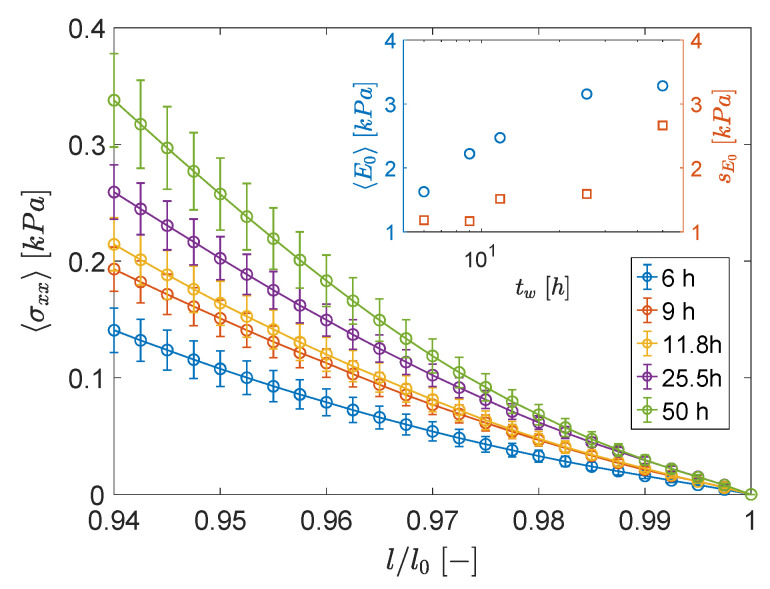
Average stress as a function of stretch ratio for samples conditioned at five different waiting times (increasing from bottom to top), as indicated. Each set of data were averaged over 12 tests. For the sake of clarity, the error-bars report only $\frac{1}{5}$ of the actual standard deviation. Inset shows the average initial compressive modulus and its actual standard deviation.

In order to have a quantitatively reliable estimation of such fluctuations, we performed tens of stress-strain tests at a fast strain rate, which is akin to probing the instantaneous elastic response of the network. Tests were carried out in an unconfined compression configuration on several samples conditioned, also in this case, at five different waiting times (i.e., 6, 9, 12, 25 and 50 h). In Figure 2 we report the average stress ($\langle \sigma_{xx}\rangle$) as a function of the stretching ratio ($l/l_{0}$). After averaging over twelve replicas, the data now show a clear trend with the waiting time: over the whole range of the investigated stretching ratio, the stress is larger for larger waiting times. However, the associated standard deviation, represented as error bars (note that their size was reduced by a factor five with respect to the actual values), is always of the same orders as that of the average stress. This behavior is fully reflected in the initial modulus (Equation (4)) obtained from the data in Figure 2 and reported in the inset: both the averages ($\langle E_{0}\rangle$) and the standard deviation ($s_{E_{0}}$) increases monotonically as a function of $t_{w}$. Thus, the overall result of the stress-strain tests confirms the presence of macroscopic fluctuations in the mechanical response.

These macroscopic elastic fluctuations suggest that only a small number of heterogeneities is present within the system, i.e., that the size of such heterogeneities was comparable or slightly smaller than the DMA gap. Indeed, large fluctuations typically arise by averaging over a small number of independent domains, since they reflect the relatively large number fluctuations among different samples. Such large heterogeneities are consistent with previous estimations of the dynamical correlation lengths up to hundreds of microns [5].

### 3.2. Dynamic Light Scattering

#### 3.2.1. Intensity Correlation Function

**Figure 3:**
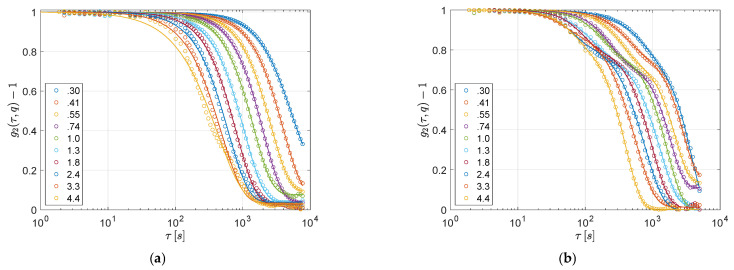
DLS measurements. Panels (**a**,**b**) report the intensity correlation function $g_{2}\left(\tau ,q\right)-1$ at different *q*-vectors for two samples, both conditioned for 6 h. From top to bottom, q varies from 0.304 to 4.4 ${\mu m}^{-1}$. Lines in panels (**a**,**b**) are single or double compressed exponential fits, respectively; see Equation (2).

To investigate the microscopic rearrangements responsible for the observed stress relaxation, we performe simultaneous measurements of DLS at a small angle. In Figure 3a,b, we present two representative examples of the normalized intensity correlation ($g_{2}\left(\tau ,q\right)-1$) as a function of delay time (τ), evaluated at different wave-vectors (q) (see Section 2.2 for details).

At all investigated wave-vector *q* values, the correlation function closely vanishes (i.e., $g_{2}\left(\tau \to \infty ,\;q\right)=1$). The few exceptions, mainly at small *q* values, are a consequence of the early shutdown of the DLS experiment (poor statistics at large times). The presence of a complete decay of the intensity correlation function indicates that the gel, at the examined length scales ($\approx \frac{2\pi }{q}$), is able to remodel its structure. The physical nature of the intermolecular bonds, that is, their ability to break and reform, allows the gel to completely rearrange at those length-scales.

Going into greater detail, the decorrelation functions show, in some cases, only one decay (Figure 3a). In four out of 10 cases, however, we observe two decays (Figure 3b) separated by a hint of an intermediate plateau. The presence of a double decay in DLS is widespread in colloidal gels and glasses both under equilibrium and out-of-equilibrium (aging) conditions. Commonly, the very early decay is ascribed to rattling within local constraints, known as β-relaxation, whereas the late one, known as α-relaxation, is related to irreversible rearrangements and is often associated with cooperative dynamics: in out-of-equilibrium systems, these events trigger irreversible structural changes and are therefore a driving mechanism for aging.

**Figure 4:**
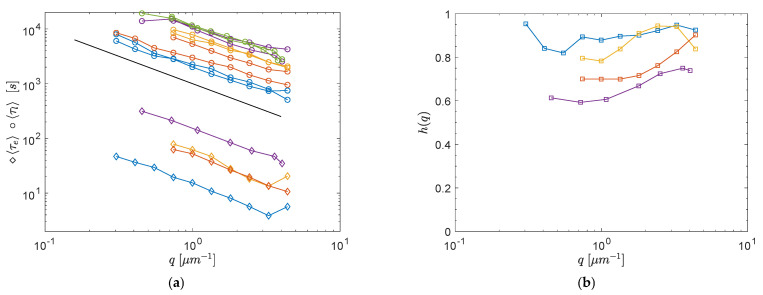
(**a**) At increasing waiting times from bottom to top, 6 (blue), 9 (red), 12 (yellow), 24 (purple) and 48 h (green): late decorrelation times (circles) vs. *q* for the two replicas. For the four samples showing a two-step decay, early decorrelation times (squares) are also reported after being divided by a factor of 10. The line is a power law with exponent −1. (**b**) Wave-vector dependence of the height of the intermediate plateau in the double step functions of Figure 3b.

The presence of a β-relaxation, however, may not be relevant to our experiments. Indeed, we speculate that even when a double step decay is observed, the first relaxation is due to irreversible rearrangements as well. The rationale behind this hypothesis is: (i) we are investigating quite large probe lengths (on the order of microns), whereas rattling within local constraints is detectable only at much smaller probe lengths; (ii) the relaxation times for the observed first decay (on the order of $10^{2}$ s) are much larger than those found for β-relaxation in similar systems [10]; (iii) both the first and the second decay are well fitted by a compressed exponential (β > 1), and the wave-vector dependence of the two relaxation times is approximately of the ballistic type, $\tau \left(q\right)\propto q^{-1}$ (see Figure 4a).

To further validate this interpretation of the first decay, in all experiments displaying a two-step relaxation, we report the height of the intermediate plateau of the field correlation function as a function of the wave-vector: h(q)=1−A(q), where A denotes the fitting parameters appearing in Equation (2b). Figure 4b shows that h(q) is always quite flat and far from being consistent with the scaling $h\left(q\right)\propto q^{-2}$ expected for the β-relaxation in physical gels [20]. This result supports the possibility that the observed two-step decays are related to the coexistence of different species of dynamic domains rather than to a fast relaxation within similar domains. Thus, we suggest that the difference between panels (a) and (b) in Figure 3 is a peculiar consequence of the existence of ultra-long DHs, which implies that only a few dynamic domains are simultaneously monitored in DLS experiments.

**Figure 5:**
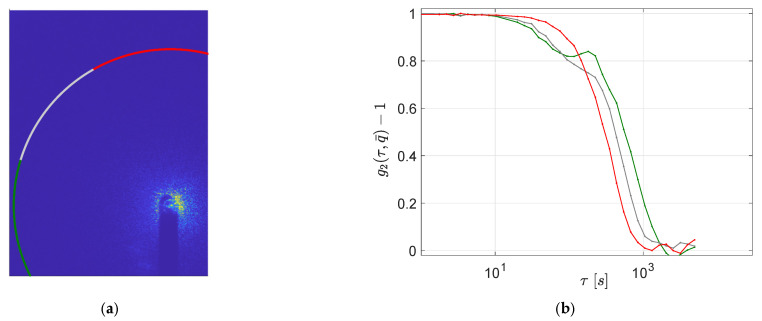
(**a**) An example of 2-D small-angle intensity scattering pattern for a sample at t_w_ = 6 h. Pixels corresponding to the same $\left|\vec{q}\right|=3.28\;\mu m^{-1}$ have been highlighted by solid lines. Different line colors correspond to the different direction ranges used to evaluate the correlation functions reported in panel (**b**) as a function of time (colors in panel b correspond to those of panel a).

To support this hypothesis, we compare the correlation functions along different *q*-vector directions. Indeed, if only few dynamic domains are simultaneously monitored in DLS, an anisotropic scatter pattern should be expected. In Figure 5a, we report the 2-D small-angle scatter pattern for a typical DLS measurement and have highlighted the pixels corresponding to a constant value of $\left|\vec{q}\right|$, for which the correlation functions have been evaluated. Three consecutive arcs of circumference were selected, corresponding to three different direction ranges. The choice of directions and arc-lengths were dictated by the limits imposed by the setup, as well as with the aim of obtaining a statistically sufficient number of pixels. The corresponding $g_{2}\left(\tau ,\vec{q}\right)-1$ functions for a sample conditioned at 6h are shown in Figure 5b. As expected, we find detectable differences among the three measured intensity correlation functions, confirming the dynamical anisotropy of the sample. We recall that the scattering volume was on the order of 1 mm^3^. Analogous features were recovered by changing the amplitude of the *q*-vector and for samples at different waiting times (see Figure S7).

#### 3.2.2. Dynamical Susceptibility

The presence of macroscopic elastic fluctuations that increase with the waiting time suggests the existence of correlations with DHs. Indeed, previous indirect estimations of the dynamical correlation lengths in the same system indicate the presence of ultra-long correlations that also increase with the waiting time in the range of 10–100 microns [5]. The same measurement for the present datasets is reported in Figure S5.

To obtain a more direct estimation of the trend of DHs with the waiting time, we quantified their behavior through the dynamic susceptibility $\chi_{4}\left(\tau ,q\right)$, which is defined at different gel ages $t_{w}$ as the variance of the time-resolved intensity correlation function $c_{I}\left(t,\tau ,q\right)$, as in [6]:

(5) $$\chi_{4}\left(\tau ,q\right)=\langle c_{I}{\left(t,\tau ,q\right)}^{2}\rangle -\langle c_{I}{\left(t,\tau ,q\right)\rangle }^{2}$$

Here the averages are calculated over the two replicas of the experiment at each $t_{w}$, as well as over the initial times t in the range monitored by DLS after each waiting time $t_{w}$. Such a time-window is reasonably small in order to consider negligible aging-induced changes in the relaxation spectra, and is sufficiently large to allow for at least decent statistics at large *q* values (where $\tau_{l}$ is relatively small), as required for the meaningful calculation of $\chi_{4}$. The dynamical susceptibility is commonly measured in simulations and experiments of gel and glasses [4,6,38,39,46,47,48], and has also recently been used to characterize other soft materials of biological and industrial interest, such as cell tissues [49,50] and inks [51]. This quantity describes the temporal evolution of dynamic heterogeneities, being roughly proportional to the volume over which the dynamics is correlated [37,52].

**Figure 6:**
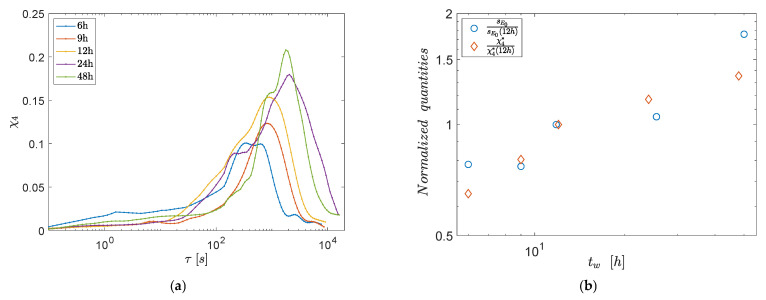
(**a**) Dynamic susceptibility $\chi_{4}\left(\tau ,q\right)$ at $q=3.3\;{\mu m}^{-1}$ and for different waiting times as indicated. (**b**) Maximum of the dynamic susceptibility $\chi_{4}^{*}$ and standard deviation of the elastic modulus $s_{E_{0}}$, both normalized by their value at $t_{w}=12\;h$, as a function of the waiting time.

We computed $\chi_{4}\left(\tau ,q\right)$ at $q=3.3\;{\mu m}^{-1}$, which is close to the upper-boundary of the investigated wave-vector range. Figure 6a shows $\chi_{4}$ as a function of time at the different investigated waiting times presented and displays the typical behavior reported for soft glassy materials, with a maximum $\chi_{4}^{*}$ at intermediate times [37,38,39,46,47,52]. $\chi_{4}^{*}$ is found to be larger for larger $t_{w}$, pointing to the growth of DHs with increasing gel ages, which is also consistent with recent results from numerical simulations [28].

Note that the growth of $\chi_{4}^{*}$ implies a reduction in the overall number of independent dynamic domains and, in turn, increasing fluctuations of the quantities that are somewhat connected with them.

In order to directly compare the relative increase in DHs and elastic fluctuations, Figure 6b shows $\chi_{4}^{*}$ and $s_{E_{0}}$ as a function of the waiting time, after normalizing both quantities for their respective value at $t_{w}=12\;h$. Both quantities increase over a similar range (about a factor of 3) over the investigated range of $t_{w}$, thus supporting the existence of a correlation between DHs and elastic fluctuations and of a close connection between DHs and EHs.

### 3.3. SEM

**Figure 7:**
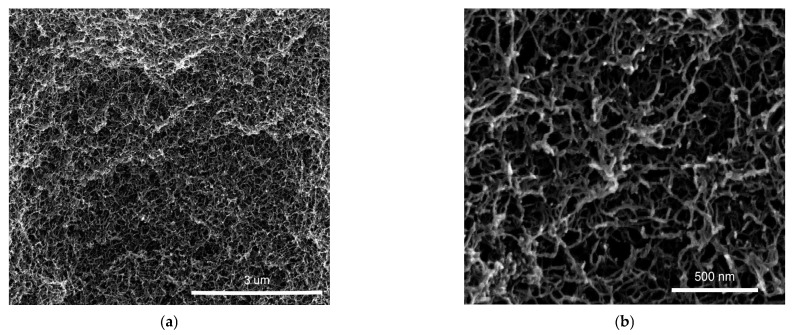
SEM of strontium alginate aerogels by sc-CO_2_ drying at different magnifications: 40,000× (**a**) and 160,000× (**b**). The average size of the pores corresponds to the characteristic length-scale obtained comparing DLS and rheology ($\lambda_{o}≃200\;\mathrm{nm}$).

Figure 7 shows two SEM pictures of this sample at different magnifications, obtained after drying the sample by means of supercritical CO_2_ extraction. For the sake of completeness, we notice that sc-CO_2_ extraction may induce some modifications of the gel network.

In spite of this, we find that the typical pore size appeared to be about a few hundred nanometers, which is fully consistent with previous SEM analysis on similar systems [30], and, remarkably, confirms an indirect estimation obtained on the same alginate gels [5]. Indeed, a comparison between DLS and rheology leads to the identification of a characteristic length-scale, $\lambda_{o}≃200\;nm$, hypothesized to be akin to a measure of the pore size in the gel [5] (the same measurement for the present datasets is reported in Figure S5). The present direct visualization by SEM seems to confirm this speculation.

## 4. Conclusions

In this manuscript, we have shown the emergence of large fluctuations in a model-system of anomalous aging gel through measurements of elasticity, stress relaxation, and multi-speckle DLS. The macroscopic nature of the observed elastic fluctuations seems to reflect the ultra-long-range nature of DHs, which is a benchmark of anomalous aging materials. Indeed, the similar increases in elastic fluctuations and dynamic susceptibility with gel age also point to the existence of correlations of DHs with elastic fluctuations.

Since both dynamic and elastic heterogeneities have a common origin in the local structure of the gels, the large observed correlations should reflect the presence of underlying large correlations in the local structure. We speculate that the relevant structural signature consists in the local degree of cross-linking, which is indeed known to be spatially heterogeneous in this type of gels [10]. Accordingly, regions exhibiting large rearrangements on the timescale of the relaxation time (DHs) should be overlapped to regions of the systems with small degrees of cross-linking. Similarly, “soft regions” characterized by small local elastic moduli (EHs) should also be tangled with a small degree of cross-linking.

As for perspective, ensemble measurements on other soft amorphous solids should clarify whether the observed large fluctuations are a general benchmark of anomalous aging gels. As a final observation, we note that gel samples are typically composed of a few macroscopic grains that are unavoidably formed during the gelation process [10]. It would be interesting to check whether the size of these grains plays the role of an upper boundary for the ultra-long correlation length of anomalous aging gels. Moreover, it is tempting to speculate that grain boundaries represent a preferential location for internal stress accumulation, somewhat similarly to what occurs with faults generating earthquakes. Intriguingly, this suggestive similarity may be carried further by considering that stress relaxation in anomalous aging gels includes avalanche-like process of subsequent events [5,15,26,27], which resemble seismic swarms.

## Supplementary Materials

The following are available online at https://www.mdpi.com/article/10.3390/polym13213618/s1. Figure S1: DLS setup, Figures S2–S5: Analysis of the stress relaxation tests, Figures S6 and S7: Evaluation of the characteristic lengths, Figure S8: Stress-strain curves at different loading rates, Figures S9 and S10 Evaluation of g_2_-1 along different wave-vector directions.
