# Supplementary material for: Elastic and Dynamic Heterogeneity in Aging Alginate Gels
Source: Polymers (Basel). 2021 Oct 20;13(21):3618. doi: 10.3390/polym13213618 (PMC8587450; doi:10.3390/polym13213618)
Supplement: Supplementary file 1 [file polymers-13-03618-s001.zip › polymers-1425863-supplementary.pdf]

Supplementary Materials

DLS Set-Up

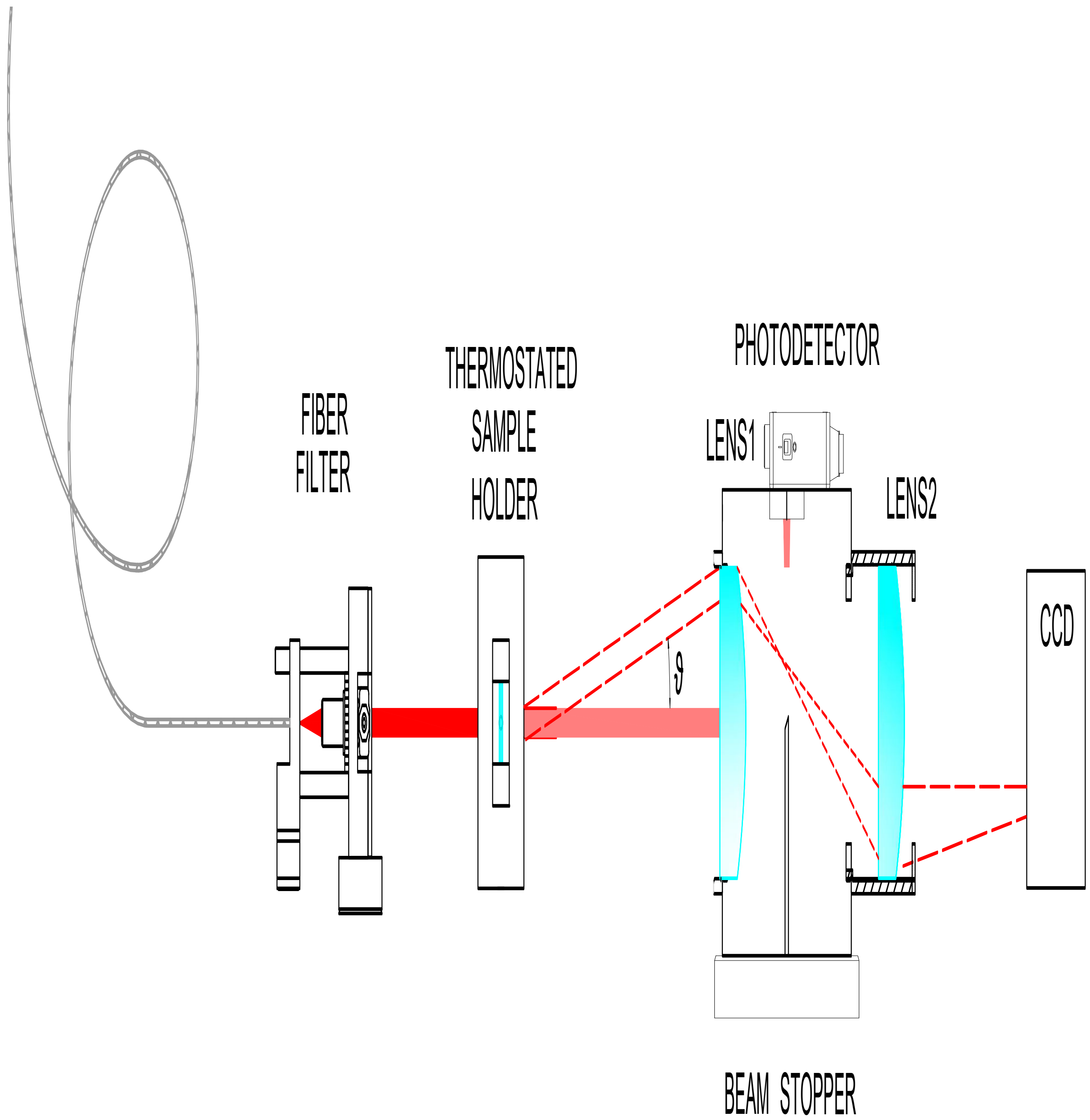

Figure S1. Small angle dynamic light scattering set-up. In brief, the laser beam (Coherent OBIS 405 nm) is spatially filtered before passing through the sample contained in a thermostated cell. Both the beam and the scattered light from the sample are collected by the L1 lens and forwarded to the CCD camera. A mirror in the focal plane of L1 prevents unscattered light from blinding the CCD sensor. Finally, another lens (L2) conjugates the focal plane of L1 with the plane of the CCD sensor.

Analysis of the Stress Relaxation Tests

To quantify the early-times behavior of the data shown in Figure 1, we fit the stress relaxation curves with the following analytical expression:

$$G(t, t_w) = G_{1s}(t_w) - \zeta(t_w) log\ (t) \quad \text{(S1)}$$

In Equation 1 $G(t, t_w)$ is the relaxation modulus that depends on the experimental time (t) and the waiting time (t_w), while G_{1s} e ζ are, respectively, the modulus evaluated at $t = 1s$ and the rate of logarithmic decay.

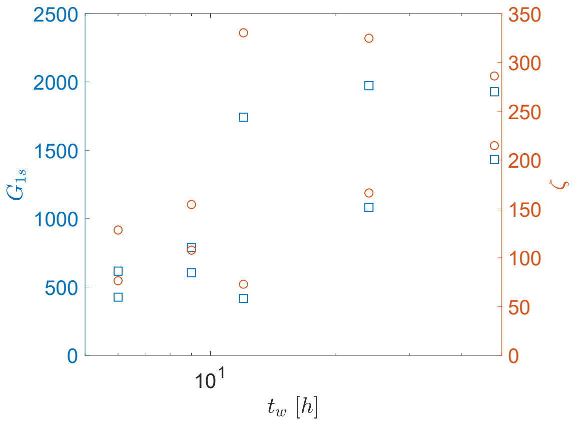

Figure S2. Values of the parameters G_{1s} (blue square), ζ (red circle) obtained from the logarithmic fitting by Equation S1.

Figure S2 shows the value of the two parameters, G_{1s} e ζ, obtained by fitting the data of Figure 1 of the main text, for times ranging between 1.2 and 12 s. As expected, the parameters are quite spread, with values that can change up to a factor of 3. As in [1], a systematic relationship between the initial modulus (represented here by $G_{1s}(t_w)$) and the rate of logarithmic decay ($\zeta(t_w)$) still persists: the bigger is G_{1s} the higher is ζ. Such relationship has led us to hypothesize that, regardless of the lack of a trend of stress relaxation curves with t_w, the causes of poor reproducibility are acting equally on both physical quantities (i.e., initial modulus and decay rate). In a simplistic way, we can state that the interpolation parameters are related to the mechanical properties of the gel as: $G_{1s}(t_w) \propto G_0(t_w)X$ and $\zeta(t_w) \propto G_0'(t_w)X$; where G_0 is the initial modulus, $G_0' \equiv dG_0/dln(t)$ the logarithmic rate, and X is a random variable equally affecting the above physical quantities. The ratio of the two model parameters ζ/G_{1s} allows to get rid of this random effect, unveil a clear monotonic trend with the waiting time (see Figure S3). The comparison of ζ/G_{1s} with the normalized weight loss from [1], $w(t)/w(0)$ suggests that the aging of strontium alginate proceeds through a gradual compactification (loss of water) and a simultaneous increase in the initial modulus G_{1s} that overcompensate the observed increase in ζ.

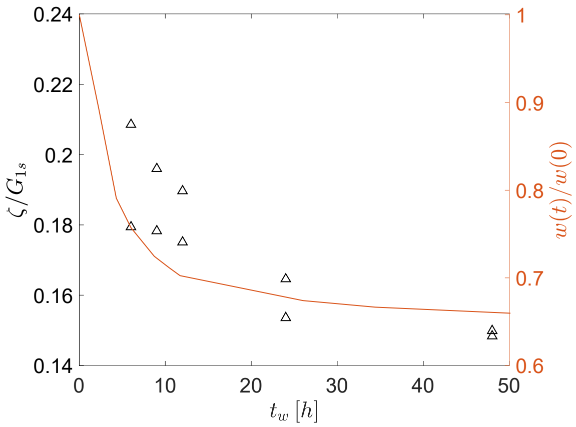

Figure S3. Ratio of the parameters ζ and G_{1s} (blue right triangles) compared with the normalized weight loss (red line).

Figure 1 in the main text also displays a lengthening in the logarithmic tract as the waiting time increases. This is likely due to an increasingly late onset of the aging-driven long-time relaxation process. The durations of these tracts are quantified by evaluating the time at which the stress relaxation curves deviate more than 3% from the logarithmic decay (Equation S1). Figure S4 shows such an onset time τ_o to be compatible with a power law of the waiting time $\tau_o \propto t_w^{\eta}$, with the exponent $\eta = 1$ being characteristic of full aging.

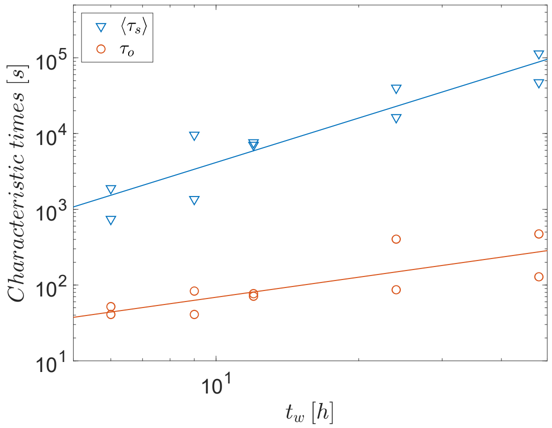

Figure S4. Characteristic times τ_o and $\langle\tau_s\rangle$ as function of t_w. Lines are indicative power law for the different datasets: τ_o and $\langle\tau_s\rangle$ with slopes 1 and 2, respectively.

Focusing on long times, $t > \tau_o$, we can verify the presence of a master curve by shifting the stress-relaxation data both horizontally (re-scaling for a characteristic time τ_s) and vertically (to take into account the variability in the modulus G_0X). All the shifted data are reported on Figure S5 showing a good overlap of the different datasets. The resulting master curve turns out to be well described by a stretched exponential function with exponent $b = 0.28$. Such a small value of the exponent b suggests that the observed late stress relaxation is a very heterogeneous process. The characteristic re-scaling time τ_s, evaluated as mean integral value (see Equation 3 in the main text), is reported in Figure S4. Despite the scattering of the shifting moduli G_s (see inset in Figure S5), the variation of $\langle\tau_s\rangle$ with t_w is compatible with a power law with exponent $\eta = 2$.

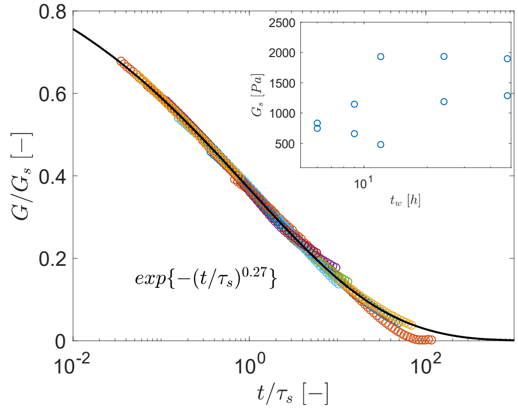

Figure S5. Data collapse of the late relaxation moduli ($t > \tau_o$); the solid line is a fitting with a stretched exponential curve ($exp\{-(t/\tau_s)^{0.28}\}$).

Evaluation of Characteristic Lengths

Following the approach of [1], we can identify two characteristic length-scales related to the rheological times τ_o and $\langle\tau_s\rangle$, by comparing them with $\tau(q)$ from DLS. We know from DLS data that the decorrelation times show a ballistic behavior:

$$\tau(q, t_w) = C(t_w)q^{-1} = C(t_w)\frac{\lambda}{2\pi} \quad \text{(S2)}$$

i.e., linear in the probe length λ. We can then define:

$$\lambda_o(t_w) \equiv \frac{2\pi\, \tau_o(t_w)}{C(t_w)}\ ; \qquad \lambda_s(t_w) \equiv \frac{2\pi\langle\tau_s(t_w)\rangle}{C(t_w)} \quad \text{(S3)}$$

In Figure S6, we report λ_o and λ_s as function of the waiting time t_w. The length $\lambda_o(t_w)$ is essentially constant, with a value of the order of 0.2 microns, whereas $\lambda_s(t_w)$ is in the range (5, 120) microns, and is fully compatible with a linearly increasing behavior.

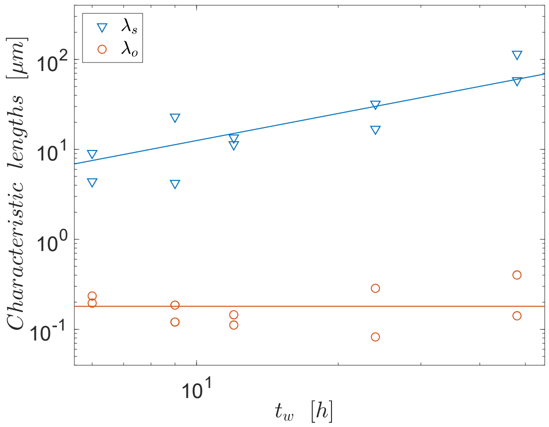

Figure S6. Characteristic length-scales λ_s and λ_o (see eq.SM3) as function of waiting time t_w. The two lines indicate a linear increase (slope 1), and a constant of 200 *nm*.

In fig. S7 we plot the normalized value of λ_s along with those of χ_4^* and s_{E_0} (already reported in the main text), in order to highlight that all these quantities increase with the waiting time over a comparable range.

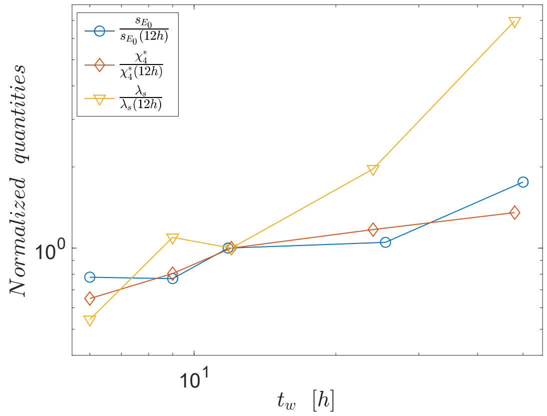

Figure S7. Standard deviation of elastic modulus s_{E_0}, maximum in the dynamic susceptibility χ_4^*, and characteristic correlation length λ_s (averaged among the two replicas), all normalized with respect to their values at 12h and plotted as a function of the waiting time t_w.

Stress-Strain Curves at Different Loading Rates

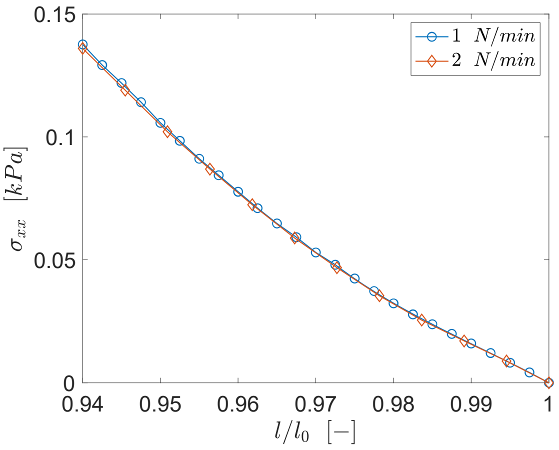

Figure S8. Two consecutive stress-strain curves carried out at different loading speeds on a sample aged for 6h. The superposition of the two curves ensures that the effect of the loading speed is null.

Evaluation of g2-1 Along Different Wave-Vector Directions

Figure S9 shows the intensity correlation function of one of the samples at tw = 6h, for which a double decay is observed. As in Figure 5 of the main text, the functions g2-1 are evaluated along three direction ranges (see Figure 5a main text). Four different values of ∣ q ∣ were analyzed, confirming that dynamic anisotropy is independent of the amplitude of the vector q.

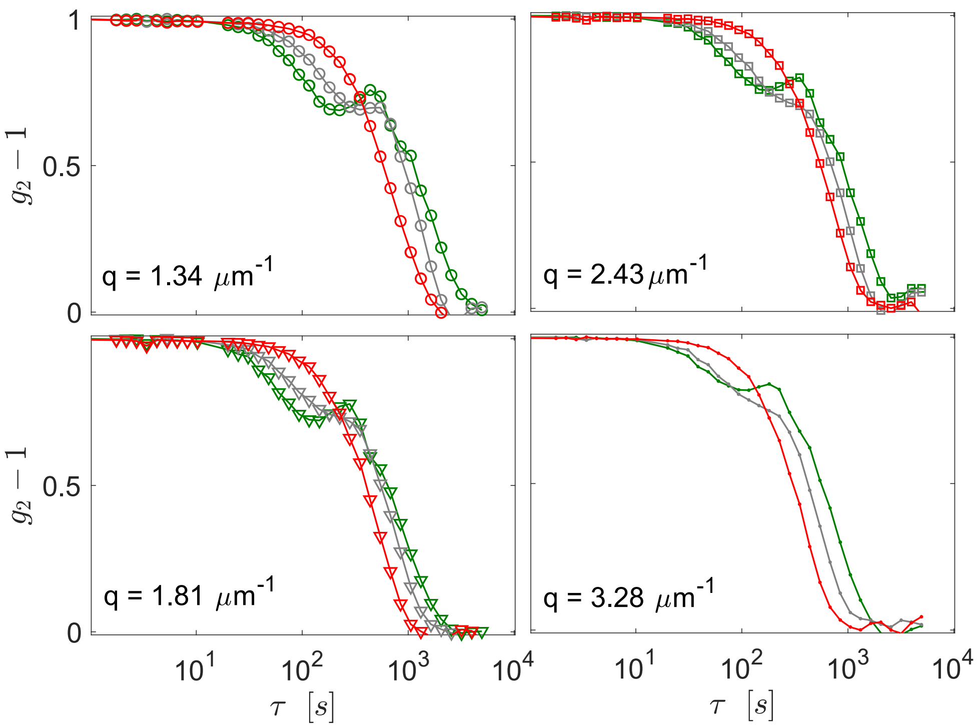

Figure S9. Intensity correlation function evaluated at four different ∣q∣ along the three different directions depicted in the Figure 5a of the main text.

Figure S10 shows the function g2-1 for one of the samples conditioned at t_w = 12h, for which only one decay is observed. Again, the different colors refer to the different directions of the q-vector depicted in Figure 5a.

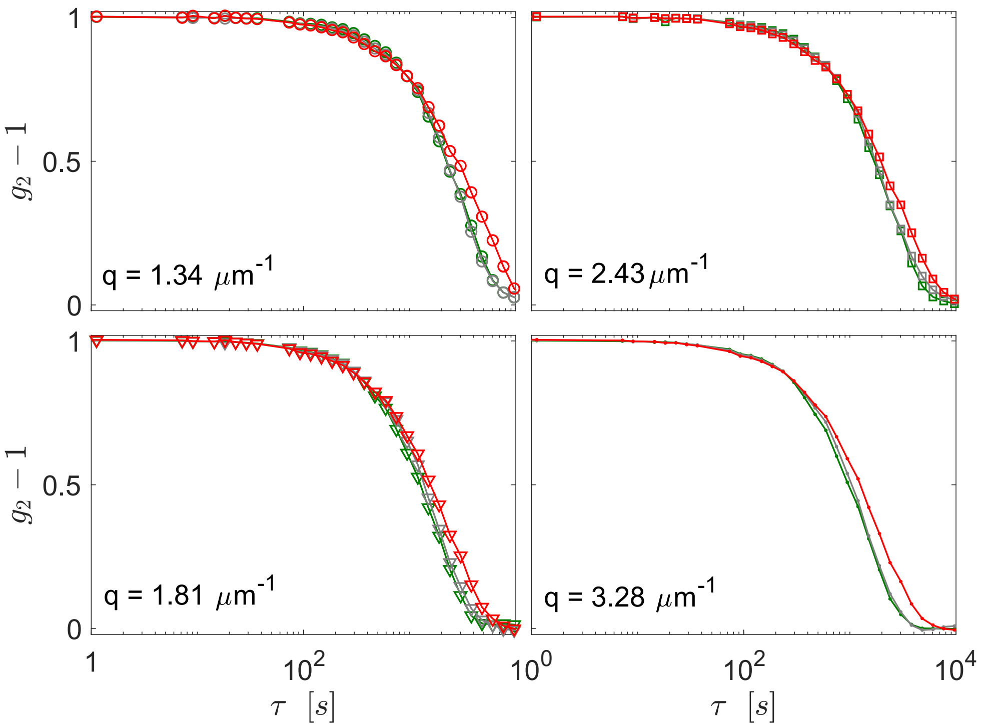

Figure S10. Same as in Figure. S9 for one of the samples conditioned at t_w=12h.

1. Pastore, R.; Siviello, C.; Greco, F.; Larobina, D. Anomalous Aging and Stress Relaxation in Macromolecular Physical Gels: The Case of Strontium Alginate. *Macromolecules* **2020**, 53 (2), 649–657.
